# An Account of a Case in Which a Foreign Body Was Lodged in the Right Bronchus

**Published:** 1843-10

**Authors:** Benjamin C. Brodie

**Affiliations:** F.R.S., etc., etc.


*An account of a case in which a Foreign Body was lodged in the Right Bronchus.*—By Sir Benjamin C. Brodie, Bart. F.R.S., etc., etc.—The author's object in this paper was to describe a case in which a half-sovereign was lodged in the right bronchus of the patient for a period of thirty days, and in which certain novel measures adopted for its removal proved successful. It was on the 3d of April, while the patient, Mr. B.,* was amusing some children, that the coin which he had in his mouth accidentally slipped into the trachea. The symptoms which succeeded were principally occasional severe fits of coughing, and a sense of pain referred to a part of the chest corresponding to the situation of the right bronchus. No particular sounds were detected by the use of the stethoscope. The patient was able to pursue his usual avocations, and made two journeys into the country. On the 19th of April, having placed himself in the prone position, with the sternum resting on a chair, and his head and neck downwards, the patient had a distinct perception of a loose body slipping forwards along the trachea; a violent convulsive cough ensued, and, on resuming the erect posture, he again had the sensation of a loose body moving in the trachea towards the chest. An apparatus of the following kind was now constructed. A platform, on which the patient could lie prone, was made to move on a hinge in the centre; so that on one end of it being elevated the other was equally depressed. On the 25th of April the patient was laid on the apparatus, with his shoulders and body fixed by means of a belt, and his head was lowered to an angle of nearly 90 degrees with the horizon. His back was then struck several times with the hand, but violent fits of choking were brought on each time, and it was not deemed prudent to continue the experiments. On the 27th it was agreed in consultation to make an opening in the trachea, between the thyroid gland

---

* Brunel, the projector and engineer of the Thames Tunnel.

and the sternum. In proposing this, the object was twofold: 1st, that an attempt might be made to extricate the coin by the forceps; 2d, that if relief could not be obtained in this manner, the artificial opening might answer the purpose of a safety-valve, and the experiment of inverting the body on the platform be repeated without the risk of causing suffocation. The operation having been performed, several attempts to extricate the coin were made, but without success; and, on each introduction of the forceps, paroxysms of convulsive coughing of such a violent kind were brought on, that it was plain that the attempts could not be persevered in without danger to life. On the 2d of May, a renewal of these trials was followed by the same results. On the 13th, the wound in the trachea having been kept from closing by the occasional introduction of a probe, the patient was placed on the moveable platform as described before; his back was then struck by the hand; two or three efforts to cough followed, and presently the patient felt the coin quit the chest, striking almost immediately afterwards, against the incisor teeth of the upper jaw, and then dropping out of the month. No spasm of the muscles of the glottis took place; a small quantity of blood was ejected at the same time, apparently coming from the granulations of the external wound. From this date the patient proceeded rapidly to get well.

The author concluded by making observations on the following heads:—1st, on the influence of the size, weight, and form of a foreign body introduced into the windpipe, in modifying the symptoms; 2d, he referred to experiments which showed that a heavy body, like the coin in the present case, was most likely to drop into the right bronchus; 3d, he adverted to the want of success attending the use of the stethoscope in this and in some other cases of the same kind; 4th, he pointed out the reasons on which he had founded his opinion, that the artificial opening made in the trachea would prevent spasm in the glottis, and thereby give greater chance of success to the experiment of inverting the patient's body on the moveable platform; lastly, he dwelt on the difficulties and dangers attending the use of the forceps, when a weighty body is lodged deeply in one of the bronchi, as was the case in his patient.—*Bullit. Med. Sci.*, from *Med. Gaz.*, July, 1843.

